# Estimation of Dopamine D_1_ Receptor Agonist Binding Kinetics Using Time-Resolved Functional Assays: Relation to Agonist-Induced Receptor Internalization by Investigational Antiparkinsonian Therapeutics

**DOI:** 10.1021/acschemneuro.5c00270

**Published:** 2025-06-19

**Authors:** Kristoffer Sahlholm, Peder Svensson, Marcus Malo, Daniel R Andersson, Nibal Betari

**Affiliations:** † Department of Medical and Translational Biology, Wallenberg Centre for Molecular Medicine, Umeå University, 901 87 Umeå, Sweden; ‡ Department of Physiology and Pharmacology, Karolinska Institutet, 171 77 Stockholm, Sweden; § Integrative Research Laboratories Sweden AB, 413 46 Gothenburg, Sweden

**Keywords:** Dopamine D1 receptor, Antiparkinsonian drugs, Binding kinetics, Half-life, pro-cognitive drugs, receptor internalization

## Abstract

The dopamine D_1_ receptor (D_1_R) is prominently expressed in the striatum and cerebral cortex and is an attractive target for treating Parkinson’s disease and cognitive impairment in schizophrenia. While newer, noncatechol D_1_R agonists such as tavapadon have shown promise in recent clinical trials, the therapeutic utility of earlier catechol agonists such as A77636 was hampered by tolerance development. The mechanism underlying tolerance induction was suggested to involve very slow A77636 dissociation from the D_1_R, promoting prominent arrestin recruitment and receptor internalization associated with delayed recycling to the cell surface. Here, we compared the signaling and binding kinetics of five D_1_R agonists—dopamine, dihydrexidine, apomorphine, A77636, and tavapadon—using two time-resolved assays of agonist-induced β-arrestin2 recruitment and G protein-coupled inward rectifier potassium (GIRK, also known as Kir3) channel activation, respectively. Additionally, D_1_R internalization was studied using cell-surface ELISA. Tavapadon and apomorphine did not induce significant D_1_R internalization, whereas pronounced internalization was observed with A77636, dopamine, and dihydrexidine. GIRK response deactivation time courses upon agonist washout were longer for A77636 and tavapadon compared to dopamine, dihydrexidine, and apomorphine. Similarly, in the β-arrestin2 assay, signal decay upon antagonist addition was slower for A77636 and tavapadon compared to the other three agonists. Tavapadon and apomorphine were partial agonists in both assays, whereas A77636 and dihydrexidine showed efficacies similar to dopamine. While our results do not provide evidence for a direct correlation between agonist dissociation and liability to tolerance induction, the possibility remains that certain combinations of agonist characteristics, such as high efficacy paired with slow dissociation, are associated with tolerance induction by D_1_R agonists.

## Graphical abstract

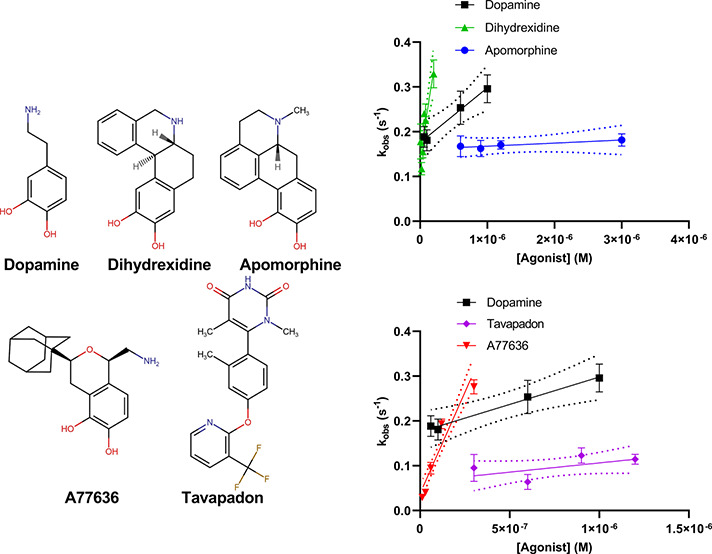

## Introduction

The dopamine D_1_ receptor (D_1_R) is a G protein-coupled receptor (GPCR) coupling predominantly to stimulatory G proteins of the G_s/olf_ type.^1,2^ It is prominently expressed in the striatum and cerebral cortex and has been implicated in voluntary motor function, reward-driven behavior, as well as in memory and cognition.^1–4^ Consistent with these physiological roles, D_1_R is a target for experimental therapeutics intended for treatment of Parkinson’s disease and cognitive impairment in schizophrenia.^1,5–7^ However, the clinical utility of earlier, high-efficacy catechol agonists such as A77636 and dihydrexidine was hampered by poor pharmacokinetic characteristics and tolerance development.^6,8^ In the case of A77636, slow dissociation of the agonist from the D_1_R, favoring prominent arrestin recruitment and receptor internalization, was suggested as the mechanism underlying tolerance induction,^9–11^ although A77636 binding kinetics have not been explored in detail. In support of this hypothesis, investigations of other GPCRs have also proposed a link between slow agonist dissociation and arrestin-receptor interactions.^12–14^ Moreover, D_1_R agonists that display low efficacy in arrestin recruitment appear less prone to induce receptor internalization.^11,15^

Drug discovery efforts directed at the D_1_R recently produced a series of novel, noncatechol ligands with improved pharmacokinetics and weak partial agonist efficacy in arrestin assays.^8^ One such noncatechol compound, tavapadon (also known as PF-06649751 and CVL-751), originally developed by Pfizer and later Cerevel Therapeutics (now part of AbbVie),^6^ recently showed promise for symptom relief in Parkinson’s disease, meeting the primary end point in a phase III study.^16^ However, there is yet little data in the public domain concerning the pharmacology of tavapadon.

Here, we aimed to compare the internalization, arrestin recruitment, and binding kinetics of five D_1_R agonists: the endogenous agonist dopamine, the experimental catechol agonists and former drug candidates A77636 and dihydrexidine, the clinically used D_1_R/dopamine D_2_ receptor (D_2_R) catechol agonist apomorphine, and tavapadon (Figure 1A). First, we used a live-cell surface enzyme-linked immunosorbent assay (ELISA) to measure internalization of D_1_R induced by exposure to each of the five D_1_R agonists. Next, to obtain kinetic data from the high-affinity, effector-bound state of the D_1_R, we used two separate, time-resolved assays of receptor activity. The first assay uses activation of G protein-coupled inward rectifier potassium (GIRK) channels, which are directly activated by G_βγ_ released from activated G protein trimers,^17^ to provide an electrophysiology-based readout of the formation and breakdown of the D_1_R - agonist - G protein complex upon agonist application and washout, whereas the second one uses nanoluciferase complementation to measure the time course of D_1_R - agonist - β-arrestin2 complex breakdown upon D_1_R antagonist addition. Finally, we compared miniG_s_ recruitment and cAMP formation induced by the D_1_R agonists.

**Figure 1:**
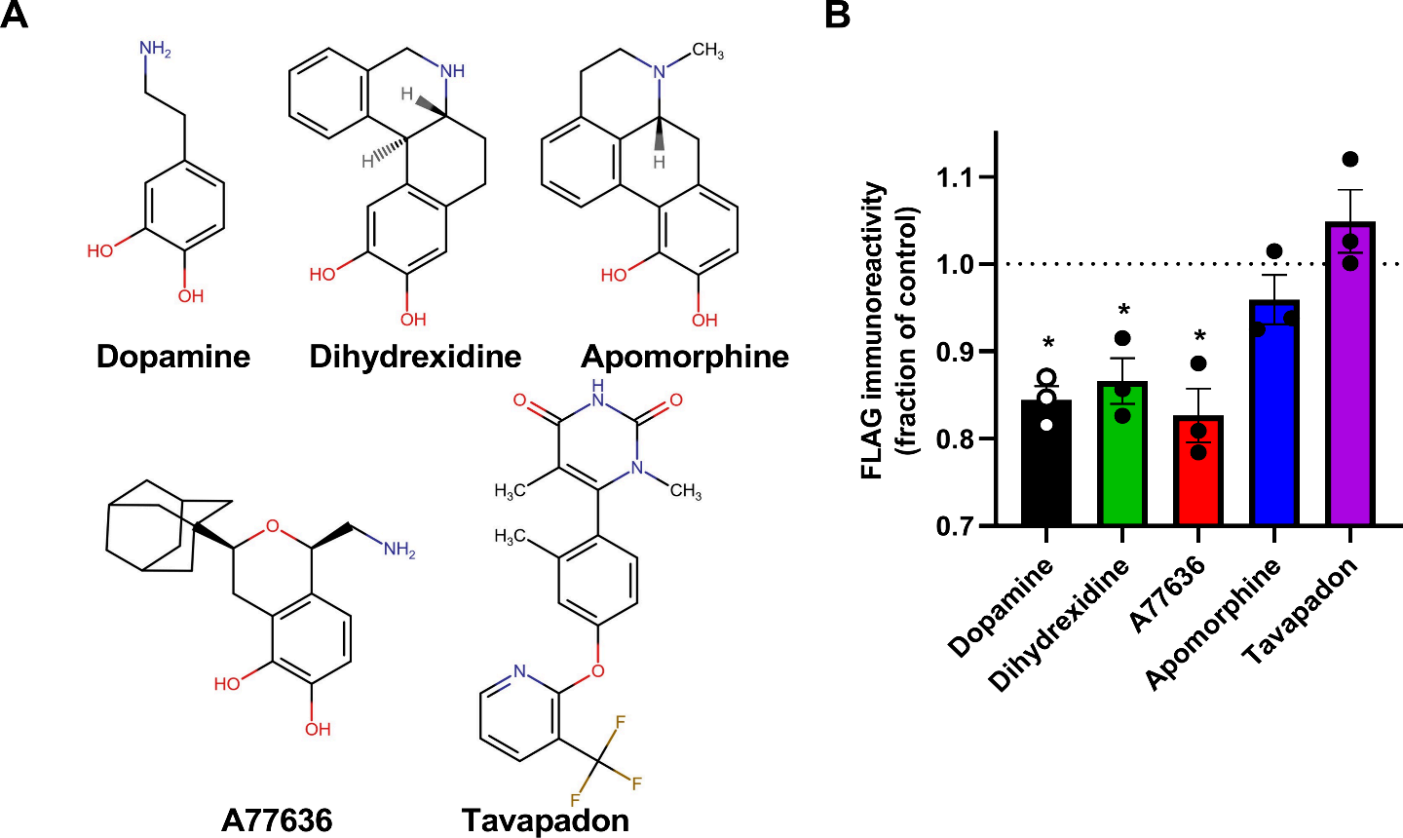
Agonist-induced D_1_R internalization. A) Structures of the D_1_R agonists used in the present study. B) Cells expressing FLAG-D_1_R-NP were incubated with 10 μM of the respective agonists or HBSS control for 1 h prior to performing the whole-cell ELISA. Bars represent means ± SEM from three separate experiments performed in octuplicate wells and superimposed dots represent means from individual experiments. Asterisks indicate statistically significant differences from 1; *, *p* < 0.05, Student’s one-sample *t* test.

## Results and Discussion

Initially, we assessed the ability of the five D_1_R agonists to induce receptor internalization by means of a live-cell surface ELISA using anti-FLAG antibodies. Thus, cells transfected with FLAG-D_1_R-NP and LgBiT-β-arrestin2 (see arrestin assay, below) were incubated with 10 μM of either agonist, or control buffer (Hank’s buffered salt solution, HBSS, supplemented with 1 mM ascorbic acid), for 1 h. Subsequently, the intact cells were assayed for FLAG immunoreactivity as a measure of D_1_R surface expression. Significant FLAG-D_1_R-NP internalization was observed upon coincubation with 10 μM A77636, dopamine, and dihydrexidine, whereas apomorphine and tavapadon did not induce appreciable loss of FLAG surface immunoreactivity (Figure 1B). To account for differences in agonist potencies, we also performed corresponding internalization experiments with the same agonists at concentrations 20 times higher than their respective EC_50_s (see EC_50_ values in Table 1) with similar results (Supplementary Figure S1).

**Table 1:** D_1_R Agonist Potencies, Estimated Forward (*k*_on_) and Reverse (*k*_off_) Rate Constants, and Kinetic *K*_d_s from the GIRK Activation Assay^a^

	pEC_50_ ± SEM (EC_50_, nM)	Efficacy relative to 30 μM dopamine ± SEM	N	k_off_ ± SEM (s^–1^)	N	k_on_ ± SEM (s^–1^ × M^–1^)	N	p*K*_d_ ± SEM
Dopamine	6.207 ± 0.033 (622)	1.022 ± 0.022	7–8	0.132 ± 0.010	7	122325 ± 37072	7–8	5.969 ± 0.090
A77636	7.382 ± 0.127 (41.5)	1.173 ± 0.119	5–10	0.025 ± 0.004	10	903422 ± 78561	4–10	7.556 ± 0.028
Dihydrexidine	7.361 ± 0.085 (43.5)	0.808 ± 0.044	3–5	0.095 ± 0.005	3	952419 ± 174431	3–5	7.002 ± 0.054
Apomorphine	5.816 ± 0.302 (1,527)	0.133 ± 0.040	4	0.090 ± 0.016	4	6910 ± 8354	4	4.883 ± 0.309
Tavapadon	6.864 ± 0.470 (137)	0.106 ± 0.023	3–12	0.027 ± 0.008	9	41157 ± 28432	7–11	6.179 ± 0.149

a pEC_50_s and efficacy values were obtained from the fits of concentration-response curves (see Methods).

In order to study D_1_R agonist binding kinetics, we first turned to the time-resolved GIRK activation assay. The time course of agonist-induced GIRK response deactivation upon agonist washout has previously been shown to reflect the rate of agonist dissociation from its receptor.^18–20^ While GIRK channels are typically activated by G_βγ_ subunits released upon activation of inhibitory G_i/o_ protein trimers, previous reports support the notion that G_βγ_ release from G_s_ proteins can also activate GIRK.^21,22^ In agreement, we recently described G_s_-dependent, dopamine-evoked opening of GIRK channels in *Xenopus* oocytes coexpressing D_1_R with GIRK1/4.^23^ Here, we employed this readout to estimate the kinetics of the five D_1_R agonists in a manner analogous to that earlier described for dopamine D_2_R agonists.^24^ Thus, GIRK current activation and deactivation rates were measured upon application and washout of agonist to obtain estimates of association (*k*_on_) and dissociation (*k*_off_) rate constants, respectively. pEC_50_s were also constructed by normalizing the response to each concentration of agonist to the response elicited by 30 μM dopamine in each respective oocyte (Figure 2A, Table 1). In oocytes expressing GIRK1/4 without D_1_R, tavapadon and apomorphine were found to block GIRK currents at 12 and 30 μM, respectively. Therefore, we limited the maximal drug concentrations used in our D_1_R-GIRK assay to 1.2 for tavapadon and 3 μM for apomorphine. For *k*_off_ estimates, the current deactivation time course following washout of an intermediately effective (i.e., close to the EC_50_) concentration of agonist was chosen, as indicated in Figure 2C and D. These concentrations were selected to ensure a clear response while simultaneously avoiding excessive agonist concentrations that could potentially favor accumulation of a lipophilic agonist in the oocyte membranes. We have earlier demonstrated that the solution exchange rate under the conditions employed here (4.5 mL min^–1^ buffer perfusion rate) is greater than 2 s^*–*1^, which is more than 10-fold faster than the fastest current deactivation rate measured here.^24^ Thus, solution exchange should not have been limiting the observed deactivation time courses. Compared to dopamine, GIRK response decay rates were considerably slower following washout of A77636 and tavapadon and slightly slower following apomorphine and dihydrexidine, reaching significance only for the former agonist. (Figure 2B, C, and D, Table 1).

**Figure 2:**
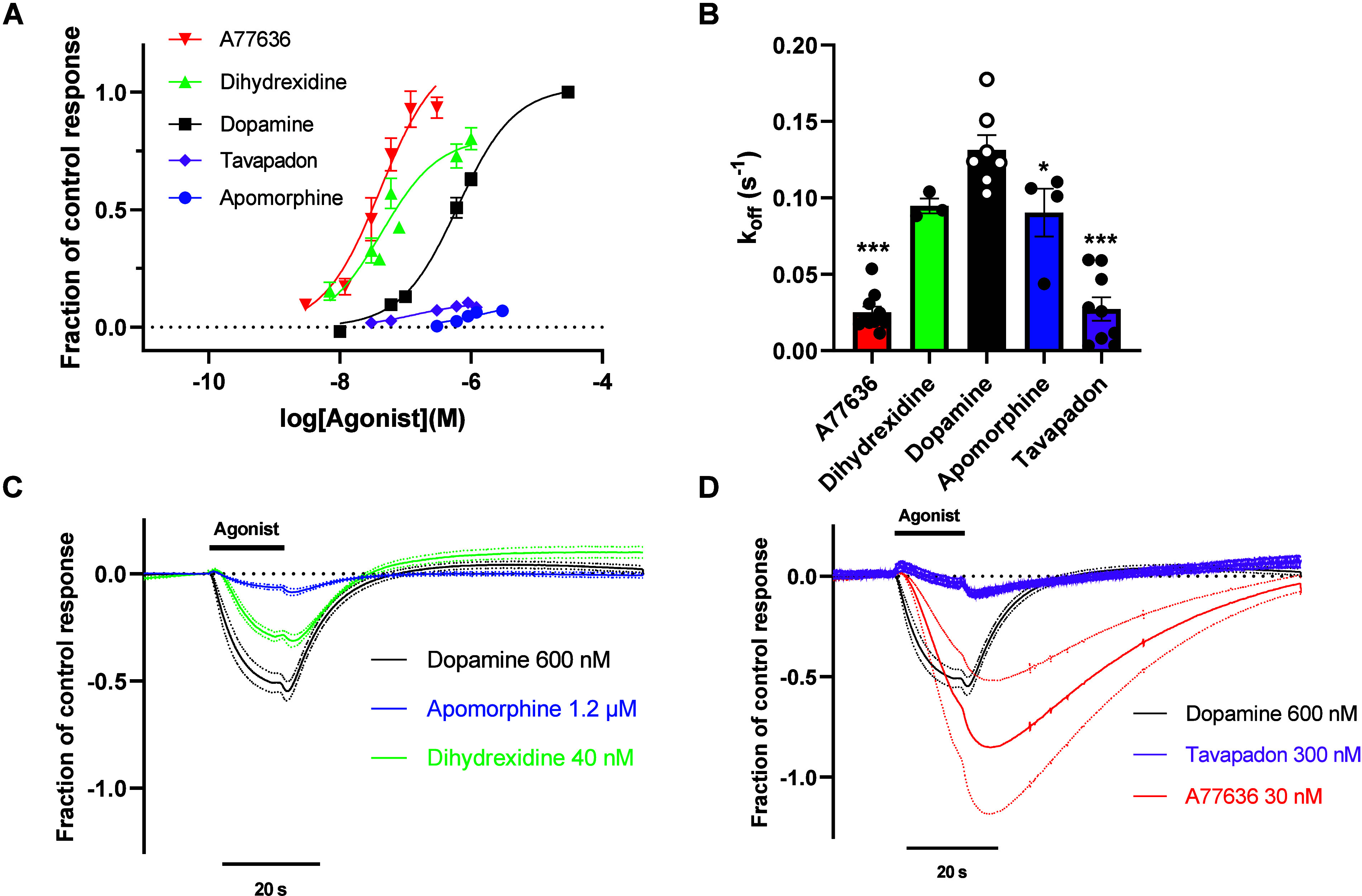
Estimation of agonist dissociation rates from D_1_R based on a GIRK activation assay. A) Concentration–response curves for agonist-induced GIRK activation in oocytes coexpressing D_1_R with GIRK1/4 channels. Data points represent means ± SEM from three to 16 separate oocytes. B) *k*_off_ values derived from the fitted τs for response decay rate following washout of subsaturating concentrations of agonist (see C and D). Bars represent means ± SEM from three to ten separate oocytes and superimposed dots represent data from individual recordings. Asterisks indicate statistically significant differences from dopamine; ***, *p* < 0.001; *, *p* < 0.05, one-way ANOVA with Dunnett’s multiple comparisons test. C, D) Averaged electrophysiology traces showing the time courses of GIRK current responses upon agonist application and washout. For clarity, apomorphine and dihydrexidine (panel C) are shown separately from A77636 and tavapadon (panel D). Averaged traces for dopamine are included in both panels for reference. Data points represent means (solid lines) ± SEM (dotted lines) from three to ten separate oocytes (same recordings as those represented in B). Before averaging, each current trace was normalized to the response to 30 μM dopamine in the same oocytes. Experiments were performed using a buffer perfusion rate of 4.5 mL min^–1^.

We previously described how plotting the rates of GIRK response activation, *k*_obs_, against varying agonist concentrations yields linear relationships, the slopes of which can be used to estimate agonist *k*_on_ at the D_2_R^24^ (see eq 2 in Methods). Here, plotting *k*_obs_ against D_1_R agonist concentrations revealed such linear relationships, which were steeper than that of dopamine for dihydrexidine and A77636, similar for tavapadon, and shallower for apomorphine, consistent with differences in *k*_on_ (Figure 3A and B, Table 1). For this analysis, we purposely used lower agonist concentrations producing *k*_obs_ below 0.4 s^–1^ in order to stay in the linear range of the relation between *k*_obs_ and agonist concentration and avoid saturation kinetics. Such saturation has been reported not only from GIRK activation assays, but also from experiments examining α_2A_ receptor activation and G protein activation by muscarinic receptors.^24–26^

**Figure 3:**
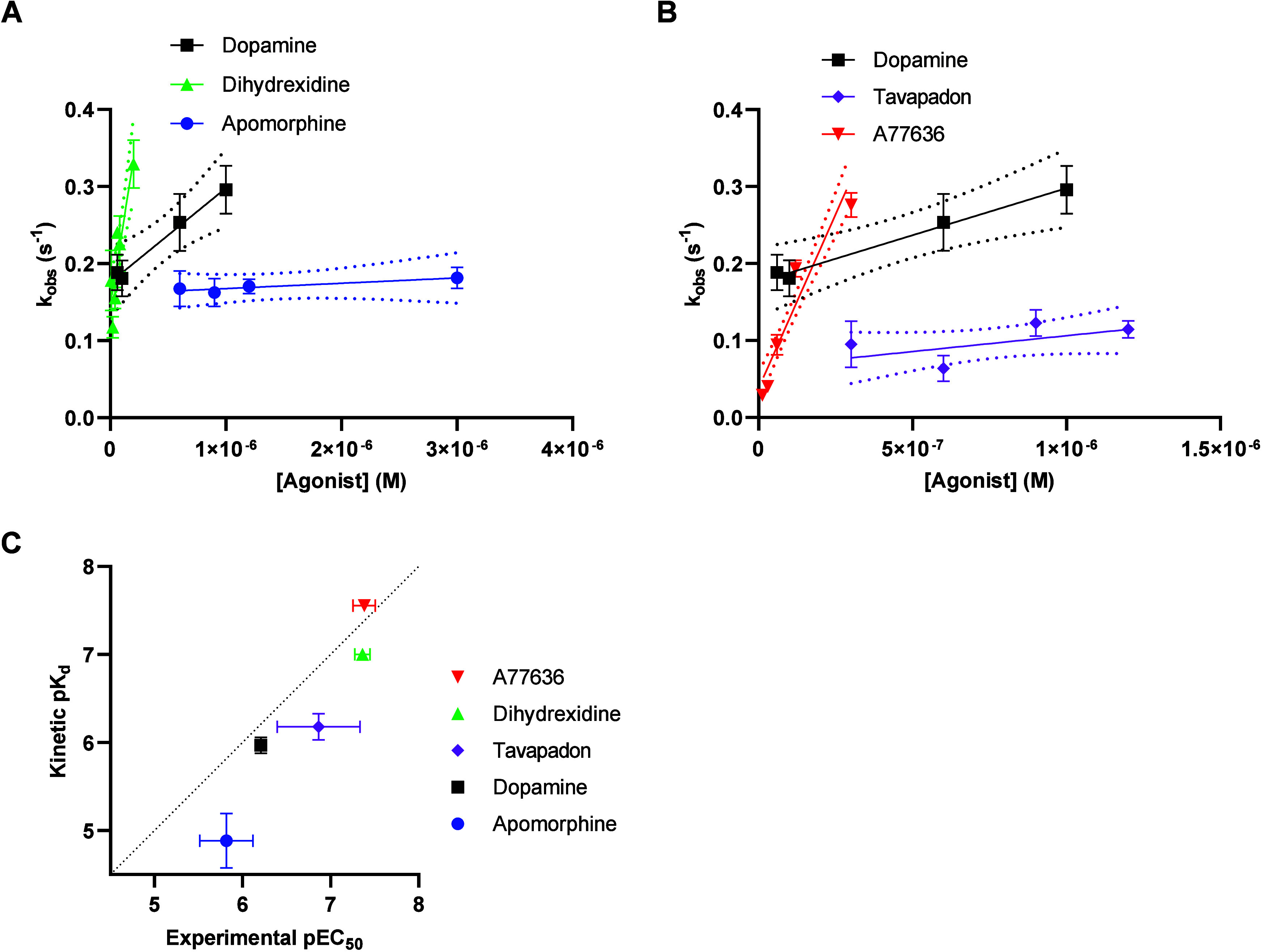
Estimation of agonist association rates at the D_1_R based on the GIRK activation assay. A, B) Varying concentrations of dopamine, dihydrexidine, apomorphine, (A) A77636, or tavapadon (B) were added to oocytes coexpressing D_1_R with GIRK1/4. The rates of rise (*k*_obs_) of the resulting current responses have been plotted against the corresponding agonist concentrations and linear fits (solid lines) and their 95% confidence bands (dotted lines) are shown. Note *x*-axis scale differences between (A) and (B). Data points represent means ± SEM from three to 11 separate oocytes. Experiments were performed using a buffer perfusion rate of 4.5 mL min^–1^. (C) Relations between kinetic p*K*_d_s, derived from *k*_off_ and *k*_on_, and pEC_50_s, obtained from the concentration–response experiments shown in Figure 2A. The correlation between pEC_50_s and kinetic p*K*_d_s for all five agonists is statistically significant (Spearman’s r = 0.9484, *p* = 0.014). Data are presented as mean ± SEM.

The *y*-axis intercept in the linear dependence of *k*_obs_ on agonist concentration can also be used to estimate *k*_off_ (see^24^ and Methods). Plotting *k*_off_ estimates derived from agonist washout experiments (Figure 2B, C and D, Table 1) against *k*_off_ estimates from the *y*-axis intercepts (Figure 3A and B) revealed a good correlation, supporting the robustness of these estimates (Supplementary Figure S2A). On the contrary, there was no obvious relation between *k*_on_ and *k*_off_ estimates (Supplementary Figure S2B), suggesting that both parameters play independent roles in determining agonist affinity. Overall, kinetic p*K*_d_s calculated for the *k*_off_ (derived from washout experiments) and *k*_on_ estimates correlated reasonably well with functional agonist potencies in the GIRK assay (Figure 3C, Table 1). However, the correlation was not as good for tavapadon and apomorphine as for dopamine, A77636, and dihydrexidine. This may be related to the smaller GIRK current responses evoked by these partial agonists and the limits imposed by GIRK channel block on their highest usable concentrations, making it more difficult to accurately estimate both potency and response kinetics, as is apparent from the larger errors associated with the pEC_50_s, *k*_on_s, *k*_off_s, and the kinetic p*K*_d_s of tavapadon and apomorphine.

Next, we aimed to corroborate our findings regarding D_1_R agonist binding kinetics using a time-resolved measure of arrestin-D_1_R interaction using a recently described nanoluciferase complementation approach.^27–29^ Thus, arrestin recruitment to the D_1_R was measured in HEK293T cells transfected with FLAG-D_1_R-NP and LgBiT-β-arrestin2 (Figure 4A, Table 2). For kinetic measurements, time-resolved luminescence was measured upon the application of a submaximally effective concentration of agonist, followed by the addition of 10 μM of the D_1_R antagonist SKF83566 ^30^ (Figure 4B-D). Again, response decay rates were slower with A77636 and tavapadon as compared to dopamine (Figure 4B, Table 2).

**Figure 4:**
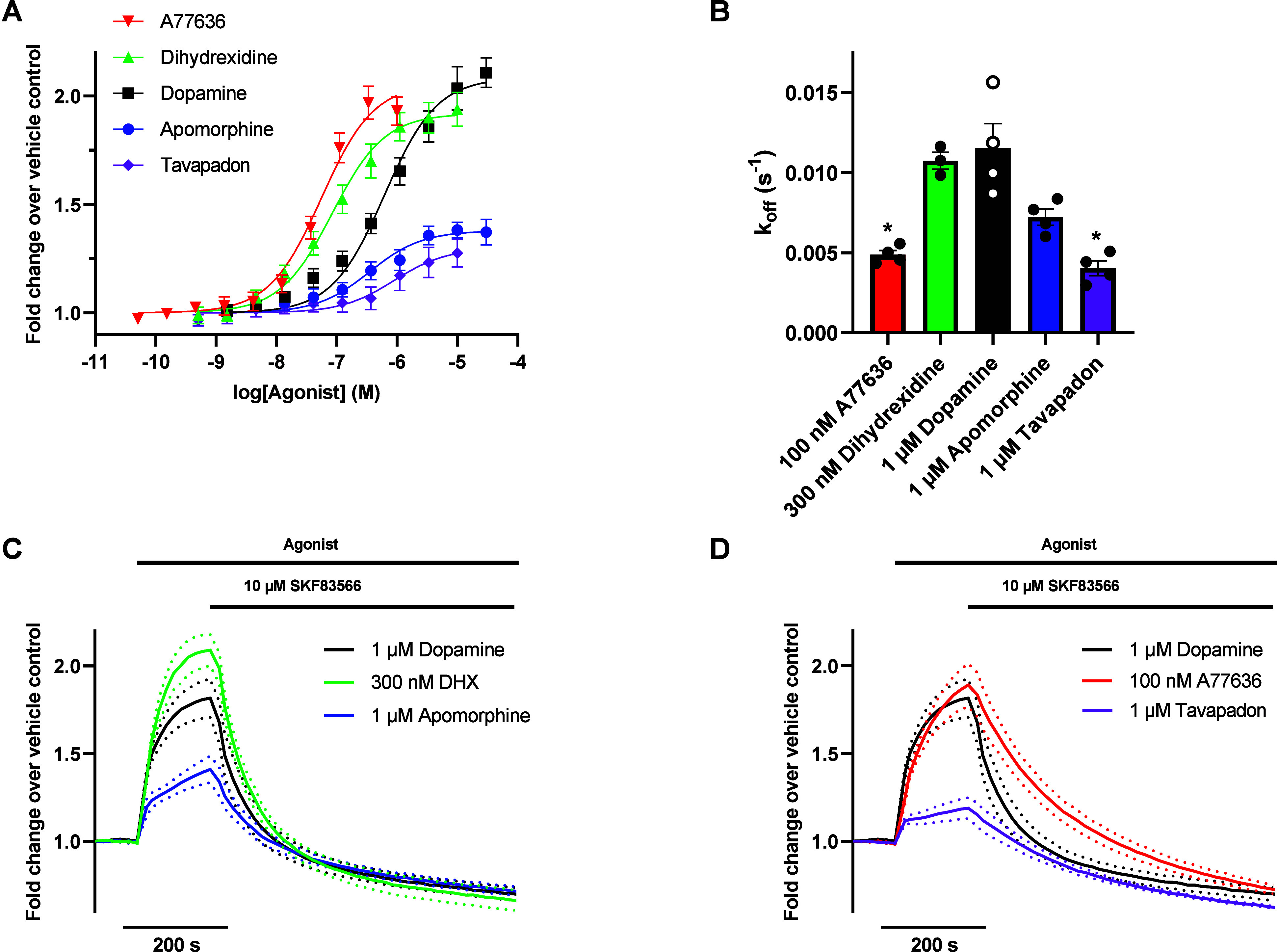
Comparison of agonist dissociation rates from D_1_R-β-arrestin2 complexes based on a nanoluciferase complementation assay. A) Concentration–response curves for agonist-induced recruitment of LgBiT-β-arrestin2 to FLAG-D_1_R-NP. Data points represent means ± SEM from five separate experiments performed in quadruplicate wells. B) *k*_off_ values derived from the fitted τs for response decay rate following SKF83566 application. Bars represent means ± SEM from three to four separate experiments performed in octuplicate wells and superimposed dots represent means from individual experiments. Asterisks indicate statistically significant differences from dopamine; *, *p* < 0.05, restricted maximum likelihood mixed-effects model with Dunnett’s multiple comparisons test. C, D) Kinetic reads showing the luminescence increase following agonist application followed by luminescence decrease after addition of the D_1_R antagonist SKF83566. For clarity, apomorphine and dihydrexidine (panel C) are shown separately from A77636 and tavapadon (panel D). Kinetic reads for dopamine are included in both panels for reference. Data points represent means (solid lines) ± SEM (dotted lines) from three to four separate experiments performed in octuplicate.

**Table 2:** Agonist Potencies, Efficacies, and Response Decay Time Constants in the Nanoluciferase Complementation FLAG-D_1_R-NP–LgBiT-β-arrestin2 Recruitment Assay^a^

	pEC_50_ ± SEM (EC_50_, nM)	Efficacy relative to dopamine ± SEM	N	Response deactivation constant ± SEM (s^–1^)	N
Dopamine	6.210 ± 0.075 (617)	1.000 ± 0.038	5	0.0115 ± 0.0015	4
A77636	7.253 ± 0.076 (55.8)	0.976 ± 0.044	5	0.0049 ± 0.0003	4
Dihydrexidine	7.074 ± 0.081 (84.4)	0.847 ± 0.031	5	0.0107 ± 0.0005	3
Apomorphine	6.455 ± 0.139 (351)	0.349 ± 0.023	5	0.0072 ± 0.0005	4
Tavapadon	6.051 ± 0.274 (890)	0.275 ± 0.049	5	0.0040 ± 0.0005	4

a pEC_50_s and efficacy values were obtained from the fits of concentration-response curves (see Methods).

Given the well-established physiological role of D_1_R in promoting adenylate cyclase activity^1,31^ and the widespread use of this effector system in previous studies of D_1_R agonists, we wanted to compare further the relative efficacies of the five D_1_R agonists in G protein-dependent signaling using both a miniG_s_ recruitment assay, analogous to the β-arrestin2 assay described above, and in a cAMP accumulation assay. Thus, HEK293T cells were transiently transfected with LgBiT-miniG_s_ and FLAG-D_1_R-NP and the agonist-dependent increase in luminescence output was measured 20 min after agonist application. The partial agonist nature of apomorphine and tavapadon was readily apparent, although the efficacies of these two ligands relative to dopamine were greater in this readout than in the GIRK and β-arrestin2 assays (Figure 5A, Table 3). Kinetic experiments revealed time courses of SKF83566-induced decay of agonist-evoked responses that were slower than in corresponding β-arrestin2 experiments. However, similar trends were observed with regards to A77636 and tavapadon kinetics, which tended to be slower than that of dopamine (Supplementary Figure S3). For cAMP measurements, HEK293T cells were transiently transfected with D_1_R and GloSensor^32^ and the cAMP-dependent signal was measured 20 min after agonist application. All five agonists elicited cAMP responses with maximal amplitudes similar to that of dopamine (Figure 5 B, Table 3).

**Figure 5:**
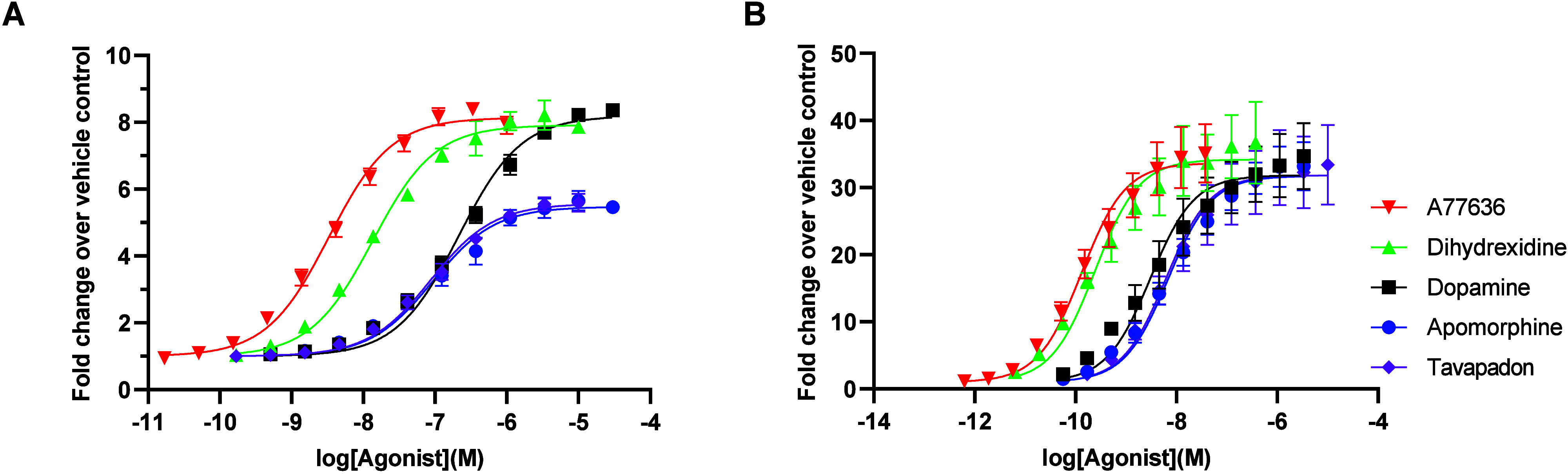
Activity of the five D_1_R agonists in LgBiT-miniG_**s**_ recruitment and GloSensor cAMP accumulation assays. Experiments were performed in HEK293T cells transiently expressing FLAG-D_1_R-NP in combination with LgBiT-miniG_s_ (A) or D_1_R with GloSensor (B). A) Concentration–response curves for agonist-induced LgBiT-miniGs recruitment. Data points represent means ± SEM of three to four independent experiments performed in quadruplicate. B) Concentration–response curves for agonist-induced cAMP accumulation. Data points represent means ± SEM of three to four independent experiments performed in quadruplicate. Data was read out 20 min after agonist addition in both A and B.

**Table 3:** Agonist Potencies, Efficacies, and Response Decay Time Constants in the Nanoluciferase Complementation FLAG-D_1_R-NP–LgBiT-miniG_s_ Recruitment and cAMP Accumulation Assays^a^

miniG_s_ recruitment				cAMP accumulation		
	pEC_50_ ± SEM (EC_50_, nM)	Efficacy relative to dopamine ± SEM	N	pEC_50_ ± SEM (EC_50_, nM)	Efficacy relative to dopamine ± SEM	N
Dopamine	6.672 ± 0.040 (213)	1.000 ± 0.018	3	8.519 ± 0.141 (3.03)	1.000 ± 0.051	4
A77636	8.480 ± 0.038 (3.31)	0.993 ± 0.016	3	9.878 ± 0.105 (0.133)	1.059 ± 0.046	4
Dihydrexidine	7.870 ± 0.045 (13.5)	0.961 ± 0.016	3	9.613 ± 0.135 (0.244)	1.076 ± 0.051	4
Apomorphine	7.035 ± 0.068 (92.2)	0.622 ± 0.017	3	8.165 ± 0.085 (6.84)	0.996 ± 0.033	4
Tavapadon	7.059 ± 0.046 (87.2)	0.635 ± 0.013	3	8.208 ± 0.148 (6.20)	0.999 ± 0.053	4

a pEC_50_s and efficacy values were obtained from the fits of concentration-response curves (see Methods).

Here, we used time-resolved signaling assays to infer binding kinetics of five D_1_R agonists. GPCRs are generally understood to exist in at least two distinct states with respect to ligand binding, having high and low affinity for agonists, respectively.^33^ The high-affinity state is thought of as representing the ternary complex of agonist, receptor, and effector (G protein or arrestin).^34,35^ Therefore, our assays likely interrogate binding kinetics relating to high-affinity binding. The notion that our functional, arguably indirect, assays captured useful information about D_1_R agonist binding kinetics at the signaling, high-affinity state is supported by the correlation between p*K*_d_ values calculated from kinetic GIRK data and experimental pEC_50_ values obtained in the GIRK and arrestin recruitment assays.

While the results of the present investigation suggest that dopamine, dihydrexidine, and apomorphine dissociate somewhat faster from the D_1_R compared to A77636 and tavapadon, they contrast sharply with earlier reports on the apparent resistance of A77636 to washout in radioligand binding and functional assays, leading some investigators to propose a very long lifetime of the D_1_R-A77636 complex.^9,10^ Conversely, the agonistic effects of A77636 were readily washed out or antagonized in our experiments. While we cannot conclusively explain these discrepancies, it is possible that the lipophilic nature of A77636 (compared to, e.g., dopamine) would allow the agonist to accumulate in cell membranes during radioligand binding experiments and rebind to the receptor after washout from the surrounding medium, thus appearing to dissociate slowly from the D_1_R itself.^36^ In our experiments, continuous buffer flow or antagonist competition could prevent rebinding (see also^37^).

Furthermore, our results suggest that slow dissociation may not in itself be indicative of a liability to internalization and tolerance induction, since this property is shared by A77636 and tavapadon while only A77636, and not tavapadon, induced D_1_R internalization. Moreover, dihydrexidine, which promotes D_1_R internalization *in vitro* (^15^ and present study), displayed the most rapid dissociation kinetics of the agonists tested here, alongside dopamine. The response decay kinetics in the arrestin assay were about 10-fold slower than in the GIRK assay, which may be related to slower equilibration upon antagonist addition to assay plate wells in the former assay, as compared to the continuous buffer flow used in the GIRK experiments. Another presumable difference between the oocyte electrophysiology experiments and the luminescence-based assays in HEK293T cells is the fact that the oocytes were voltage-clamped at – 80 mV, whereas HEK293T cells typically have a resting potential of ∼−25 mV.^38^ We previously reported that depolarization to 0 mV decreased dopamine potency compared to – 80 mV and increased the rate of GIRK response termination upon washout,^23^ thus producing an effect opposite to the difference observed here between electrophysiology and luminescence-based readouts. In any case, the relative differences in response termination kinetics, with A77636 and tavapadon being slower than dopamine, found in the GIRK assay were reflected in the arrestin assay, thus strengthening our conclusions. While *k*_on_ estimates differed up to 140-fold between agonists (dihydrexidine vs apomorphine) and tended to be higher for the internalization-inducing agonists, the *k*_on_ of the noninternalizing tavapadon differed from that of dopamine by only 3-fold. We therefore find it difficult to see a compelling link between agonist *k*_on_ and D_1_R internalization based on the present data.

A recent study presented evidence that slow agonist dissociation at the angiotensin II type 1 receptor favors receptor-arrestin interactions specifically after receptor internalization, within the endosome, leading to delayed recycling of the receptor to the cell surface.^39^ Indeed, a previous study suggested that A77636 may produce tolerance by a similar mechanism.^9^ Whatever the mechanism might be at the endosomal level, the present results suggest that A77636 dissociation is not as slow as has been previously suggested and that somewhat slower dissociation does not necessarily predispose to receptor internalization, as exemplified by tavapadon, although the possibility remains that slow dissociation paired with high efficacy, as with A77636, could play a role in tolerance induction. On the other hand, it seems natural to associate the absence of significant D_1_R internalization observed here with tavapadon and apomorphine with their weak partial agonism, particularly in the β-arrestin2 readout. Arrestin recruitment is generally considered to be an important first step in GPCR internalization since arrestin-bound GPCRs are frequently targeted to clathrin-coated pits, which go on to form endocytic vesicles.^40^ The present results are congruent with other studies that linked the propensities of D_1_R agonists to induce receptor internalization to their relative efficacies in recruiting arrestins, and which reported low efficacies of both tavapadon and apomorphine in such recruitment.^11,15,41–43^

We note that, in our GIRK activation readout, the efficacy of tavapadon relative to dopamine is considerably lower than what has been previously described for this ligand in likewise G protein-dependent cAMP accumulation experiments by Teng et al.^44^ and Martini et al.,^42^ who both described tavapadon as a G protein-biased agonist. However, a recent study using bioluminescence resonance energy transfer (BRET)-based sensors of G protein activation showed that this agonist is a partial agonist at G_s_, and a full agonist at G_olf_.^45^ Meanwhile, the relative efficacy of tavapadon in our arrestin recruitment assay is close to that reported by Teng et al.^44^ and Martini et al.,^42^ who used nanoluciferase complementation and BRET assays, respectively, to report on β-arrestin recruitment to the D_1_R. Similarly, the efficacy of apomorphine in the GIRK activation assay was lower than what has been reported from cAMP assays, although a partial agonist efficacy of ∼75% was noted by Conroy et al.^15,41^ We would speculate that the low efficacies of these two ligands in GIRK activation reflect their partial agonism in G_s_ coupling, in combination with low signal amplification in the GIRK assay. As described by Touhara and MacKinnon,^22^ GIRK channel opening by G_s_ is relatively inefficient due to the low concentrations of free G_βγ_ generated by the slow cycling of this G protein subtype as compared to G_i/o_ proteins.

On the other hand, we replicated the strong efficacies of tavapadon and apomorphine in our cAMP assay. cAMP-dependent readouts and other second messenger assays are prone to amplification,^46^ and we therefore consider that these efficacy differences may be the result of a receptor reserve in the cAMP assays (here and in previous reports). On the contrary, readouts proximal to receptor activation, such as “direct” (i.e., not gene synthesis-dependent) arrestin and miniG recruitment assays, and GIRK activation, generally have a low tendency for signal amplification.^47,48^ This notion is also supported by the GIRK and arrestin recruitment assay EC_50_s for DHX, A77636, and dopamine being close to their respective affinities as reported from radioligand binding studies,^10,49,50^ whereas the cAMP EC_50_s in our study were orders of magnitude lower.

EC_50_s in the miniG_s_ assay were intermediate, somewhat lower than in the GIRK and arrestin assays, but higher than in the cAMP assay. Likewise, the relative efficacies of tavapadon and apomorphine were higher than in the GIRK and arrestin assays, while being lower compared to the cAMP assay. These findings may suggest the existence of some receptor reserve in the miniGs assay despite its “proximal” nature, as has been described in the literature,^51^ or it might represent an artifact of using a miniG protein. Notably, miniG proteins are engineered to mimic nucleotide-empty G proteins and were originally designed to stabilize the ternary complex of receptor, agonist, and effector (miniG).^52^ While recruitment assays using miniG proteins typically offer a higher signal-to-background ratio compared to assays using native-like, full-size G proteins, these artificial constructs may distort some aspects of native receptor function and an increased lifetime of agonist-induced receptor-miniG_s_ complexes (compared to complexes containing full-length G_s_) upon antagonist addition has been reported for the β2-adrenergic receptor.^51,53^ In agreement, we observed much slower deactivation kinetics for all five agonists in the miniG_s_ recruitment assay than in the β-arrestin2 and GIRK activation assays, although tavapadon and (at a trend level) A77636 were once again slower than dopamine (Supplementary Figure S3). Finally, we cannot rule out the presence of a certain degree of “bias” or functional selectivity among the studied agonists, such that they may preferentially activate one signaling pathway over another.^54^ Indeed, several noncatechol agonists such as tavapadon have been reported to preferentially activate G protein-dependent pathways over arrestins, a behavior which has been suggested to underlie the low liability to *in vivo* tolerance induction by these compounds.^6,8^ In addition, tavapadon was recently shown to be biased toward G_olf_ over G_s_ signaling.^45^

The clinical efficacy of tavapadon in patients with Parkinson’s disease suggests that partial D_1_R agonism is sufficient to evoke a physiologically relevant stimulus in the living brain. In this context it is interesting to note that experimental evidence points to the existence of a receptor reserve for D_1_R-mediated cAMP accumulation also in native tissue, at least in the rat striatum.^55^ Meanwhile, partial agonism at the level of arrestin recruitment may be linked to a low propensity to induce D_1_R internalization and tolerance, as has been previously suggested.^11,15^ Thus, G protein bias and/or downstream amplification of some (e.g., G protein-dependent cAMP signaling) signaling pathways but not others (β-arrestin-dependent internalization) may be the mechanism behind the clinically favorable profile of partial D_1_R agonists.^6,8,15^ Finally, it should be emphasized that the *in vitro* environments of our investigations do not fully recapitulate the native environment of the D_1_R, such as that found in neurons *in vivo*.

## Conclusions

Here, we used functional assays to deduce novel information about the receptor binding kinetics of five D_1_R agonists. This information and the time-resolved experiments presented here may be useful for future studies of these and related ligands. This strategy can likely also be applied to the comparison of agonist binding kinetics at other receptor subtypes that can be studied in time-resolved live-cell signaling assays. In addition, our results suggest that the dissociation rate of a D_1_R agonist is not intrinsically linked to β-arrestin2 recruitment efficacy nor to receptor internalization. However, we cannot exclude the possibility that slow dissociation can be relevant to desensitization and tolerance induction under certain conditions, for examples when paired with high agonist efficacy, as is the case with A77636.

## Methods

### Molecular Biology

For oocyte electrophysiology experiments, cDNA encoding the human D_1_R (from Dr. Marc Caron, Duke University, NC) was in pcDNA3.1 whereas GIRK1 and GIRK4 (synthesized by GenScript, inc., Piscataway, NJ) were in pXOOM (provided by Dr. Søren-Peter Olesen, University of Copenhagen, Denmark). Plasmids were linearized using XhoI, followed by *in vitro* transcription using the T7 mMessage mMachine kit (Ambion, Austin, TX, USA). cRNA concentration and purity were determined by spectrophotometry.

The FLAG-D_1_R-NP (’native peptide’) construct was synthesized by GenScript (Piscataway, NJ) on a pcDNA3.1+ backbone (Thermo Fisher Scientific, Waltham, MA) and designed similarly to a D_1_R construct previously described by Laschet et al.^28^ Thus, an N-terminal cleavable influenza hemagglutinin signal peptide (KTIIALSYIFCLVFA), which promotes cell surface expression, followed by a FLAG tag (DYKDDDDK) was added to the N-terminus of the human D_1_R protein, while a linker (GNSGSSGGGGSGGGGSSG) followed by an NP tag (GVTGWRLCERILA) were added to the C-terminus. The LgBiT-βarr2 construct (in pNBe3; Promega, Madison, WI) consists of rat βarr2 N-terminally fused to LgBiT;

VFTLE­DFVGD­WEQTA­AYNLD­QVLEQ­GGVSS­LLQNL­AVSVT­PIQRIV­RSGEN­ALKIDI­HVIIP­YEGLS­ADQMA­QIEEV­FKVV­YPVDD­HHFK­VILPY­GTLVI­DGVTP­NMLN­YFGR­PYEGI­AVFDG­KKITV­TGTLW­NGNK­IIDER­LITPD­GSMLF­RVTINS, and was a gift from Drs. Julien Hanson and Céline Laschet (University of Liège, Belgium). The LgBiT-miniG_s_ construct uses a GGGGS linker to fuse LgBiT N-terminally to miniG_s__393 (previously described by Nehmé et al.^52^) and was synthesized by GenScript and cloned into pcDNA3.1. Untagged human D_1_R was cloned into pXOOM by GenScript.

### Ligands

A77636 was obtained from Cayman Chemicals (Ann Arbor, MI), dihydrexidine and tavapadon were purchased from MedChem Express (Monmouth Junction, NJ), and dopamine and apomorphine were from Sigma-Aldrich (St. Louis, MO, USA). SKF83566 was acquired from Axon Medchem (Groningen, The Netherlands). Ligands were dissolved in DMSO or, in the case of dopamine, water and diluted in buffer to the desired final concentrations. The maximum final concentration of DMSO used in any experiment was 0.1% v/v.

### Oocyte Preparation and Electrophysiology

Oocytes from the African clawed toad, *Xenopus laevis*, were purchased from EcoCyte Bioscience (Castrop-Rauxel, Germany). Oocytes were injected with 50 nL water containing 8.5 ng D_1_R cRNA and 40 pg of each GIRK1 and GIRK4 cRNA using the Nanoject III (Drummond Scientific, Broomall, PA, USA). Injected cells were incubated for 6 days at 12 °C in modified Barth’s solution (MBS), composed of (in mM): 88 NaCl, 1 KCl, 2.4 NaHCO_3_, 15 HEPES, 0.33 Ca­(NO_3_)­2, 0.41 CaCl_2_, 0.92 MgSO_4_, and 2.5 sodium pyruvate, supplemented with 25 U/mL penicillin and 25 μg/mL streptomycin and adjusted to pH 7.6 with NaOH. Electrophysiology recordings were performed at 22 °C using the eight-channel, two-electrode voltage-clamp OpusXpress 6000A (Molecular Devices, San José, CA, USA).^56,57^ Continuous perfusion was maintained at 4.5 mL min^–1^. Data were acquired at membrane potentials of – 80 mV and sampled at 156 Hz using the OpusXpress 1.10.42 (Molecular Devices) software. A high-potassium extracellular buffer was used in order to increase the inward rectifier potassium channel current at negative potentials (in mM: 64 NaCl, 25 KCl, 0.8 MgCl_2_, 0.4 CaCl_2_, 15 HEPES, and 1 ascorbic acid, adjusted to pH 7.4 with NaOH), yielding a K^+^ reversal potential of about −40 mV. Ascorbic acid was included to prevent spontaneous oxidation of D_1_R agonists. For construction of concentration–response curves, the peak response elicited by each concentration of an agonist was normalized to the response to 30 μM dopamine, evoking a maximal D_1_R-mediated response, in that same oocyte. Oocytes were selected for electrophysiology recordings based on having holding currents at −40 mV of less than 0.5 μA. Similarly, recordings in which holding currents at −40 mV were greater than 0.5 μA after data acquisition at −80 mV were discarded.

### Luciferase Complementation Assays

We employed an earlier described nanoluciferase assay^27,28^ to measure the interaction between D_1_R and β-arrestin2 or miniG_s_. HEK293T cells (a gift from Dr. Jonathan Gilthorpe, Umeå University, Sweden) were grown at 37 °C with 5% CO_2_ in 10 cm culture dishes (VWR part of Avantor, Radnor, PA) containing Dulbecco’s modified eagle medium (DMEM; Thermo Fisher Scientific) supplemented with 0.01% penicillin/streptomycin (Thermo Fisher Scientific) and 10% FBS (Thermo Fisher Scientific). Cells were transfected with 1 μg/dish FLAG-D_1_R-NP and 1 μg/dish LgBiT-βarr2 or LgBiT-miniG_s_ using linear polyethylenimine (PEI; Polysciences Inc., Valley Road Warrington, PA. Empty plasmid vector (pcDNA3.1+) was added to the transfection mixture to bring the total amount of transfected plasmid to 20 μg/dish. Cells were transfected at 70–80% confluency, at which point old medium was aspirated and replaced with 5 mL of fresh DMEM (supplemented as above). The transfection mixture was added, and cells were left in the incubator for 4 h.

For concentration–response curve generation, the medium of the dish was changed to fresh, complete DMEM and cells were left to grow for another 24 h after transfection. The following day, cells were trypsinated and centrifuged for pellet recovery before resuspension in HBSS (Bio-West, Nuaillé, France) supplemented with 1 mM ascorbic acid. Cells were counted using a TC20 automated cell counter (Bio-Rad, Hercules, CA) and further diluted to 500,000 cells ml^–1^ in HBSS with 1 mM ascorbic acid and 2 μM of the nanoluciferase substrate coelenterazine 400a (Nanolight Technology, Pinetop, AZ). 100 μL cell solution was pipeted into each well of a white flat-bottom 96-well plate (Thermo Fisher Scientific). Serial dilutions of D_1_R agonist (in HBSS containing 1 mM ascorbic acid) were added to the 96-well plate, using 4 wells per agonist concentration and a control column receiving only HBSS. The plate was incubated at room temperature for 15 or 20 min prior to luminescence measurement in a TriStar2 LB 942 multimode reader (Berthold Technologies, Bad Wildbad, Germany) with an integration time of 10 ms.

For kinetic measurements, cells were trypsinated directly after the 4-h transfection, pelleted, resuspended in complete DMEM, counted, further diluted in DMEM to 250,000 cells ml^–1^ and seeded into a poly d-lysine-coated white 96-well plate at a density of 25,000 cells/well, and incubated overnight. The following day, DMEM was replaced with HBSS (100 μL per well) containing 1 mM ascorbic acid and 2 μM coelenterazine 400a. In these kinetic experiments, luminescence was recorded from each well each 14 s for 1 h with an integration time of 5 ms. Following a baseline read of 81 s, 10 μL of agonist dissolved in HBSS containing 1 mM ascorbic acid was injected into each well to result in a final, submaximally effective concentration as indicated in Figure 4C and D. After another 140 s, 10 μL of SKF83566, dissolved in DMSO and diluted in HBSS, was injected to yield a final concentration of 10 μM.

### Live-Cell Enzyme-Linked Immunosorbent Assay (ELISA)

Cell surface expression of D_1_R was evaluated essentially as described earlier for related GPCRs.^27,58^ In brief, following transfection (as described above), cells were trypsinated, resuspended in complete DMEM (supplemented as above), counted, further diluted in DMEM, and replated at 50,000 cells per well in a volume of 100 μL in transparent 96-well plates (Thermo Fischer Scientific) previously coated with poly-d-lysine (Sigma-Aldrich) and returned to the incubator for further growth and adhesion. After another 30–48 h incubation when the cells were at least 80% confluent, DMEM was aspirated and replaced with HBSS supplemented with 1 mM ascorbic acid and 10 μM of either D_1_R agonist or control. After 1 h incubation with D_1_R agonists at 37 °C, wells were washed twice with 100 μL per well of chilled (4 °C) phosphate-buffered saline (PBS; VWR part of Avantor) containing 0.5% (w/v) bovine serum albumin (BSA; Sigma-Aldrich). After washing, cells were incubated with 50 μL per well of horseradish peroxidase-linked mouse anti-FLAG M2 antibody (A8592; Sigma-Aldrich) diluted 1/20,000 with chilled PBS containing 1% BSA. Following 1 h incubation at 4 °C, well contents were aspirated and washed four times with chilled PBS containing 0.5% BSA. Next, 50 μL per well of the horseradish peroxidase substrate 3,3′,5,5′-Tetramethylbenzidine (T0440; Sigma-Aldrich) were added and plates were incubated at 37 °C for 20 min. Finally, 50 μL per well of 2 M HCl were added to each well and absorbance was measured at 450 nm in a Berthold TriStar2 LB 942 plate reader.

### cAMP Assay

HEK293T cells were grown in 10 cm dishes and transfected as described above with 6 μg GloSensor 22F plasmid (Promega, Madison, WI) and 1 μg D_1_R plasmid in pXOOM, together with empty pcDNA3.1 as needed to reach 20 μg plasmid DNA per dish. The cAMP assay was performed essentially as previously described.^32,57^ In brief, 24 h after transfection, cells were lifted off, pelleted, and resuspended counted and further diluted to 500,000 cells ml^–1^ in HBSS supplemented with 300 μM 3-isobutyl-1-methylxanthine (IBMX; Sigma-Aldrich) and GloSensor reagent (Promega) and 1 mM ascorbic acid. Cells were dispensed into white 96-well plates (ThermoFischer Scientific) at a density of 50,000 cells per well and left to equilibrate at room temperature for 1 h, followed by agonist addition. Twenty min later, luminescence intensity was read in a Berthold Tristar 2S plate reader with a 1-s integration time.

### Data Analysis

Agonist concentration–response relationships were analyzed by fitting sigmoidal functions using nonlinear regression in GraphPad Prism 8 (GraphPad Software, San Diego, CA, USA). The following equation was used for fitting:

$$Y=\mathrm{Bottom}+\left(\mathrm{Top}-\mathrm{Bottom}\right)/\left(1+10^{\left({\mathrm{LogEC}}_{50}-X\right)}\right)$$ 1

where *Y* is the response normalized either to the response to 30 μM dopamine (GIRK experiments), or to the fitted maximal response to dopamine (β-arrestin2 and miniG_s_ recruitment experiments and cAMP accumulation experiments), Top is the maximal response of the agonist in question, and *X* is the logarithm of agonist concentration. Bottom is the baseline response and was constrained to either 0 (GIRK activation experiments) or 1 (β-arrestin2 and miniG_s_ recruitment experiments as well as cAMP accumulation experiments).

Electrophysiology concentration–response data was initially processed in Clampfit 10.6 (Molecular Devices) by subtracting the basal (agonist-independent) current and quantifying the current amplitude evoked by each concentration of agonist.

Data are reported as mean ± SEM throughout the manuscript. In experiments measuring response decay following agonist washout or antagonist addition, monoexponential functions were fitted to the relevant intervals of individual traces corresponding to response decay following agonist washout (see the Results and Discussion), outputting the deactivation time constant, τ deact. Estimates of the dissociation rate constant, *k*_off_, were obtained as 1/τdeact.

Association rate constant estimates were derived from recordings of GIRK current activation in response to applications of various agonist concentrations. Monoexponential functions were fit to cover 80% of the current increase in response to agonist using Levenberg–Marquardt least-squares fitting in Clampfit 10, outputting the activation time constant, τ act. The observed activation rate (*k*_obs_) was defined as 1/τ act, and subsequently used for estimation of the association rate constant as the slope of the dependence of *k*_obs_ on agonist concentration, over the range of concentrations where this relation was linear (see ref 24), using the following relation:

2 $$k_{\mathrm{obs}}=\left[A\right]\times k_{\mathrm{on}}+k_{\mathrm{off}}$$

where [*A*] is the agonist concentration, *k*_on_ the association rate constant, and *k*_off_ the dissociation rate. However, instead of using Equation 2, *k*_off_ was calculated separately from the response decay time constant upon agonist washout, τ deact, as described above.

Kinetic *K*_d_ values were calculated as

3 $$K_{d}=k_{\mathrm{off}}/k_{\mathrm{on}}$$

Statistical analysis was performed using GraphPad Prism 8, using *p* < 0.05 as the significance limit.

## Supplementary Material



## Notes

Additional graphs of agonist-induced internalization and kinetic data obtained from GIRK and miniGs experiments (PDF)

## Abbreviations

D_1_R: dopamine D_1_ receptor
DMEM: Dulbecco’s modified eagle medium
ELISA: enzyme-linked immunosorbent assay
GIRK: G protein-coupled inward rectifier potassium channel
HBSS: Hank’s buffered salt solution
MBS: modified Barth’s solution
NP: native peptide

## References

[1] Jones-Tabah J., Mohammad H., Paulus E. G., Clarke P. B. S., Hébert T. E. (2022). The Signaling
and Pharmacology of the Dopamine D1 Receptor. Front Cell Neurosci.

[2] Beaulieu J. M., Espinoza S., Gainetdinov R. R. (2015). Dopamine
receptors - IUPHAR Review
13. Br. J. Pharmacol..

[3] Arnsten A. F. (2011). Catecholamine
influences on dorsolateral prefrontal cortical networks. Biol. Psychiatry.

[4] Enriquez-Traba J., Arenivar M., Yarur-Castillo H. E., Noh C., Flores R. J., Weil T., Roy S., Usdin T. B., LaGamma C. T., Wang H. (2025). Dissociable control
of motivation and reinforcement
by distinct ventral striatal dopamine receptors. Nat. Neurosci.

[5] Rajagopal L., Huang M., Mahjour S., Ryan C., Elzokaky A., Svensson K. A., Meltzer H. Y. (2024). The dopamine
D1 receptor positive
allosteric modulator, DETQ, improves cognition and social interaction
in aged mice and enhances cortical and hippocampal acetylcholine efflux. Behav Brain Res..

[6] Bezard E., Gray D., Kozak R., Leoni M., Combs C., Duvvuri S. (2024). Rationale and Development of Tavapadon,
a D1/D5-Selective
Partial Dopamine Agonist for the Treatment of Parkinson’s Disease. CNS Neurol Disord Drug Targets.

[7] Abi-Dargham A., Javitch J. A., Slifstein M., Anticevic A., Calkins M. E., Cho Y. T., Fonteneau C., Gil R., Girgis R., Gur R. E. (2022). Dopamine D1R Receptor
Stimulation as a Mechanistic Pro-cognitive Target for Schizophrenia. Schizophr Bull..

[8] Gray D. L., Allen J. A., Mente S., O’Connor R. E., DeMarco G. J., Efremov I., Tierney P., Volfson D., Davoren J., Guilmette E. (2018). Impaired β-arrestin
recruitment and reduced desensitization by non-catechol agonists of
the D1 dopamine receptor. Nat. Commun..

[9] Ryman-Rasmussen J. P., Griffith A., Oloff S., Vaidehi N., Brown J. T., Goddard W. A., Mailman R. B. (2007). Functional
selectivity of dopamine
D1 receptor agonists in regulating the fate of internalized receptors. Neuropharmacology.

[10] Lin C. W., Bianchi B. R., Miller T. R., Stashko M. A., Wang S. S., Curzon P., Bednarz L., Asin K. E., Britton D. R. (1996). Persistent
activation of the dopamine D1 receptor contributes to prolonged receptor
desensitization: studies with A-77636. J. Pharmacol
Exp Ther.

[11] Nilson, A. N. ; Felsing, D. E. ; Wang, P. ; Jain, M. ; Zhou, J. ; Allen, J. Functionally selective dopamine D1 receptor endocytosis and signaling by catechol and non-catechol agonists. bioRxiv 2024 10.1101/2024.04.15.589637.40111449

[12] Wacker D., Wang S., McCorvy J. D., Betz R. M., Venkatakrishnan A. J., Levit A., Lansu K., Schools Z. L., Che T., Nichols D. E. (2017). Crystal Structure of an LSD-Bound Human
Serotonin
Receptor. Cell.

[13] Bech E. M., Kaiser A., Bellmann-Sickert K., Nielsen S. S., Sørensen K. K., Elster L., Hatzakis N., Pedersen S. L., Beck-Sickinger A. G., Jensen K. J. (2019). Half-Life Extending
Modifications of Peptide YY. Mol. Pharmaceutics.

[14] Jones B., Buenaventura T., Kanda N., Chabosseau P., Owen B. M., Scott R., Goldin R., Angkathunyakul N., Corrêa I. R., Bosco D. (2018). Targeting GLP-1 receptor
trafficking to improve agonist efficacy. Nat.
Commun..

[15] Conroy J. L., Free R. B., Sibley D. R. (2015). Identification of G protein-biased
agonists that fail to recruit β-arrestin or promote internalization
of the D1 dopamine receptor. ACS Chem. Neurosci..

[16] https://news.abbvie.com/2024-09-26-AbbVie-Announces-Positive-Topline-Results-from-Phase-3-TEMPO-1-Trial-Evaluating-Tavapadon-as-a-Monotherapy-for-Parkinsons-Disease. Accessed 2024-12-11.

[17] Dascal N., Kahanovitch U. (2015). The Roles
of Gβγ and Gα in Gating
and Regulation of GIRK Channels. Int. Rev. Neurobiol.

[18] Benians A., Leaney J. L., Tinker A. (2003). Agonist unbinding
from receptor dictates
the nature of deactivation kinetics of G protein-gated K+ channels. Proc. Natl. Acad. Sci. U. S. A..

[19] Bünemann M., Bücheler M. M., Philipp M., Lohse M. J., Hein L. (2001). Activation
and deactivation kinetics of alpha 2A- and alpha 2C-adrenergic receptor-activated
G protein-activated inwardly rectifying K+ channel currents. J. Biol. Chem..

[20] Tauber M., Ben-Chaim Y. (2023). Functional
consequences of a rare human serotonergic
5-HT. Front Pharmacol.

[21] Hatcher-Solis C., Fribourg M., Spyridaki K., Younkin J., Ellaithy A., Xiang G., Liapakis G., Gonzalez-Maeso J., Zhang H., Cui M. (2014). G protein-coupled
receptor
signaling to Kir channels in Xenopus oocytes. Curr. Pharm. Biotechnol.

[22] Touhara, K. K. ; MacKinnon, R. Molecular basis of signaling specificity between GIRK channels and GPCRs. Elife 2018, 7 10.7554/eLife.42908. PMC6335053 30526853

[23] Ågren R., Sahlholm K. (2020). Voltage-Dependent Dopamine
Potency at D. Front Pharmacol.

[24] Ågren, R. ; Stepniewski, T. M. ; Zeberg, H. ; Selent, J. ; Sahlholm, K. Dopamine D _2_ Receptor Agonist Binding Kinetics-Role of a Conserved Serine Residue. Int. J. Mol. Sci. 2021, 22 (8) 10.3390/ijms22084078. PMC8071183 33920848

[25] Ilyaskina O. S., Lemoine H., Bünemann M. (2018). Lifetime of muscarinic receptor-G-protein
complexes determines coupling efficiency and G-protein subtype selectivity. Proc. Natl. Acad. Sci. U. S. A..

[26] Vilardaga J. P., Steinmeyer R., Harms G. S., Lohse M. J. (2005). Molecular basis
of inverse agonism in a G protein-coupled receptor. Nat. Chem. Biol..

[27] Burström V., Ågren R., Betari N., Valle-León M., Garro-Martínez E., Ciruela F., Sahlholm K. (2023). Dopamine-induced
arrestin recruitment and desensitization of the dopamine D4 receptor
is regulated by G protein-coupled receptor kinase-2. Front Pharmacol.

[28] Laschet C., Dupuis N., Hanson J. (2019). A dynamic and screening-compatible
nanoluciferase-based complementation assay enables profiling of individual
GPCR-G protein interactions. J. Biol. Chem..

[29] Burström V., Xu K., Garro-Martínez E., Mach R. H., Sahlholm K., Betari N. (2025). A nanoluciferase complementation-based assay for monitoring
β-arrestin2 recruitment to the dopamine D. Biochem Biophys Rep.

[30] Kaiser C., Jain T. (1985). Dopamine receptors: functions, subtypes
and emerging concepts. Med. Res. Rev..

[31] Stoof J. C., Kebabian J. W. (1981). Opposing roles for D-1 and D-2 dopamine receptors in
efflux of cyclic AMP from rat neostriatum. Nature.

[32] Gilissen J., Geubelle P., Dupuis N., Laschet C., Pirotte B., Hanson J. (2015). Forskolin-free cAMP
assay for Gi-coupled receptors. Biochem. Pharmacol..

[33] De
Lean A., Stadel J. M., Lefkowitz R. J. (1980). A ternary complex model explains
the agonist-specific binding properties of the adenylate cyclase-coupled
beta-adrenergic receptor. J. Biol. Chem..

[34] Shalgunov V., van Waarde A., Booij J., Michel M. C., Dierckx R. A. J. O., Elsinga P. H. (2019). Hunting for the high-affinity state of G-protein-coupled
receptors with agonist tracers: Theoretical and practical considerations
for positron emission tomography imaging. Med.
Res. Rev..

[35] Gurevich V. V., Pals-Rylaarsdam R., Benovic J. L., Hosey M. M., Onorato J. J. (1997). Agonist-receptor-arrestin,
an alternative ternary complex with high agonist affinity. J. Biol. Chem..

[36] Packeu A., Wennerberg M., Balendran A., Vauquelin G. (2010). Estimation
of the dissociation rate of unlabelled ligand-receptor complexes by
a ’two-step’ competition binding approach. Br. J. Pharmacol..

[37] Sahlholm K., Zeberg H., Nilsson J., Ögren S. O., Fuxe K., Århem P. (2016). The fast-off
hypothesis revisited:
A functional kinetic study of antipsychotic antagonism of the dopamine
D2 receptor. Eur. Neuropsychopharmacol.

[38] Kirkton R. D., Bursac N. (2011). Engineering biosynthetic excitable tissues from unexcitable
cells for electrophysiological and cell therapy studies. Nat. Commun..

[39] Tóth A. D., Szalai B., Kovács O. T., Garger D., Prokop S., Soltész-Katona E., Balla A., Inoue A., Várnai P., Turu G. (2024). G protein-coupled receptor
endocytosis generates spatiotemporal bias in β-arrestin signaling. Sci. Signal.

[40] Moo E. V., van Senten J. R., Bräuner-Osborne H., Møller T. C. (2021). Arrestin-Dependent
and -Independent Internalization of G Protein-Coupled Receptors: Methods,
Mechanisms, and Implications on Cell Signaling. Mol. Pharmacol..

[41] Ryman-Rasmussen J. P., Nichols D. E., Mailman R. B. (2005). Differential activation
of adenylate
cyclase and receptor internalization by novel dopamine D1 receptor
agonists. Mol. Pharmacol..

[42] Martini M. L., Ray C., Yu X., Liu J., Pogorelov V. M., Wetsel W. C., Huang X. P., McCorvy J. D., Caron M. G., Jin J. (2019). Designing Functionally Selective
Noncatechol Dopamine D. ACS Chem. Neurosci..

[43] Park H., Urs A. N., Zimmerman J., Liu C., Wang Q., Urs N. M. (2020). Structure-Functional-Selectivity Relationship Studies
of Novel Apomorphine Analogs to Develop D1R/D2R Biased Ligands. ACS Med. Chem. Lett..

[44] Teng X., Chen S., Nie Y., Xiao P., Yu X., Shao Z., Zheng S. (2022). Ligand recognition
and biased agonism
of the D1 dopamine receptor. Nat. Commun..

[45] Nguyen A. M., Semeano A., Quach V., Inoue A., Nichols D. E., Yano H. (2025). Characterization of
Gα. iScience.

[46] Smith J. S., Lefkowitz R. J., Rajagopal S. (2018). Biased signalling: from simple switches
to allosteric microprocessors. Nat. Rev. Drug
Discov.

[47] Gillis, A. ; Gondin, A. B. ; Kliewer, A. ; Sanchez, J. ; Lim, H. D. ; Alamein, C. ; Manandhar, P. ; Santiago, M. ; Fritzwanker, S. ; Schmiedel, F. ; Low intrinsic efficacy for G protein activation can explain the improved side effect profiles of new opioid agonists. Sci. Signal 2020, 13 (625) 10.1126/scisignal.aaz3140. 32234959

[48] Hoare S. R. J., Tewson P. H., Sachdev S., Connor M., Hughes T. E., Quinn A. M. (2022). Quantifying the
Kinetics of Signaling and Arrestin
Recruitment by Nervous System G-Protein Coupled Receptors. Front Cell Neurosci.

[49] Lovenberg T. W., Brewster W. K., Mottola D. M., Lee R. C., Riggs R. M., Nichols D. E., Lewis M. H., Mailman R. B. (1989). Dihydrexidine,
a
novel selective high potency full dopamine D-1 receptor agonist. Eur. J. Pharmacol..

[50] Mottola D. M., Laiter S., Watts V. J., Tropsha A., Wyrick S. D., Nichols D. E., Mailman R. B. (1996). Conformational
analysis of D1 dopamine
receptor agonists: pharmacophore assessment and receptor mapping. J. Med. Chem..

[51] Wan Q., Okashah N., Inoue A., Nehmé R., Carpenter B., Tate C. G., Lambert N. A. (2018). Mini G
protein probes
for active G protein-coupled receptors (GPCRs) in live cells. J. Biol. Chem..

[52] Nehmé R., Carpenter B., Singhal A., Strege A., Edwards P. C., White C. F., Du H., Grisshammer R., Tate C. G. (2017). Mini-G proteins: Novel tools for
studying GPCRs in
their active conformation. PLoS One.

[53] Manchanda Y., ElEid L., Oqua A. I., Ramchunder Z., Choi J., Shchepinova M. M., Rutter G. A., Inoue A., Tate E. W., Jones B. (2024). Engineered mini-G proteins
block the internalization of cognate GPCRs and disrupt downstream
intracellular signaling. Sci. Signal.

[54] Urban J. D., Clarke W. P., von Zastrow M., Nichols D. E., Kobilka B., Weinstein H., Javitch J. A., Roth B. L., Christopoulos A., Sexton P. M. (2007). Functional selectivity and classical concepts
of quantitative pharmacology. J. Pharmacol Exp
Ther.

[55] Battaglia G., Norman A. B., Hess E. J., Creese I. (1986). Functional recovery
of D1 dopamine receptor-mediated stimulation of rat striatal adenylate
cyclase activity following irreversible receptor modification by N-ethoxycarbonyl-2-ethoxy-1,2-dihydroquinoline
(EEDQ): evidence for spare receptors. Neurosci.
Lett..

[56] Papke R. L., Stokes C. (2010). Working with OpusXpress: methods
for high volume oocyte
experiments. Methods.

[57] Ågren R., Betari N., Saarinen M., Zeberg H., Svenningsson P., Sahlholm K. (2023). In Vitro Comparison
of Ulotaront (SEP-363856) and Ralmitaront
(RO6889450): Two TAAR1 Agonist Candidate Antipsychotics. Int. J. Neuropsychopharmacol.

[58] Kozielewicz P., Turku A., Bowin C. F., Petersen J., Valnohova J., Cañizal M. C. A., Ono Y., Inoue A., Hoffmann C., Schulte G. (2020). Structural
insight into small molecule action on Frizzleds. Nat. Commun..

