# Supplementary material for: Estimation of Dopamine D1 Receptor Agonist Binding Kinetics Using Time-Resolved Functional Assays: Relation to Agonist-Induced Receptor Internalization by Investigational Antiparkinsonian Therapeutics
Source: ACS Chem Neurosci. 2025 Jun 19;16(13):2502–12. doi: 10.1021/acschemneuro.5c00270 (PMC12232300; doi:10.1021/acschemneuro.5c00270)
Supplement: Supplementary file 1 [file cn5c00270_si_001.pdf]

Estimation of Dopamine D_1 Receptor Agonist Binding Kinetics Using Time-Resolved Functional Assays – Relation to Agonist-Induced Receptor Internalization by Investigational Antiparkinsonian Therapeutics

Kristoffer Sahlholm[1, 2], Peder Svensson[3], Marcus Malo[3], Daniel R Andersson[3], Nibal Betari[1]

[1] Department of Medical and Translational Biology, Wallenberg Centre for Molecular Medicine, Umeå University, Umeå, Sweden

[2]Department of Physiology and Pharmacology, Karolinska Institutet, Stockholm, Sweden

[3] Integrative Research Laboratories Sweden AB, Gothenburg, Sweden

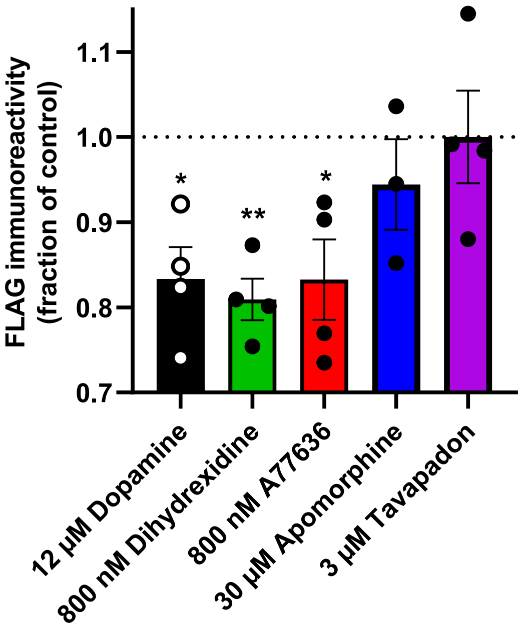

Supplementary Figure S1. Cells expressing FLAG-D_1R-NP were incubated with the indicated concentrations of the respective agonists or HBSS control for 1 h prior to performing the whole-cell ELISA. Agonist concentrations were chosen to be ~20 times higher than the EC50s measured in the GIRK activation assay (see Table 1). Bars represent means ± SEM from three to four separate experiments performed in octuplicate wells and superimposed dots represent means from individual experiments. Asterisks indicate statistically significant differences from 1; **, $p < 0.01$; *, $p < 0.05$, Student's one-sample t-test.

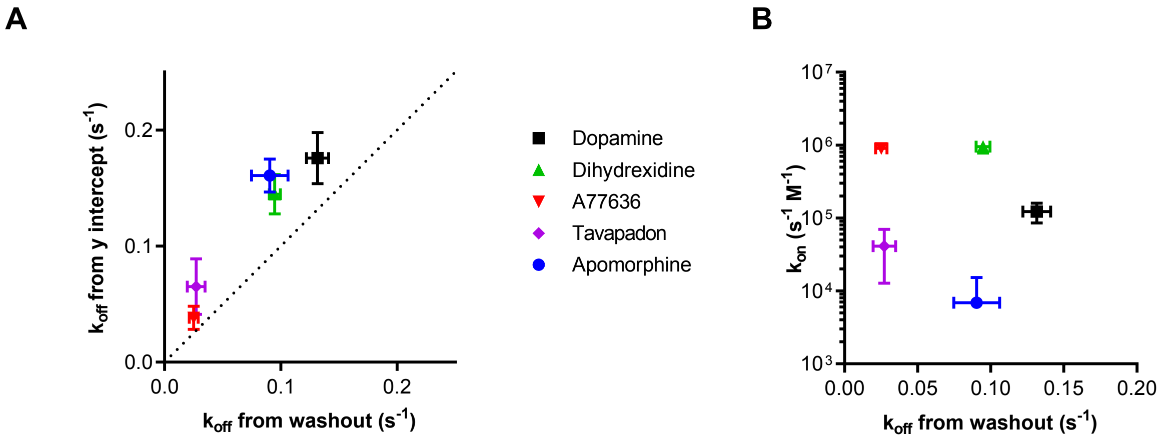

Supplementary Figure S2. (A) Correlation between k_{off} estimates obtained from the y-axis intercept of linear fits to the relation between k_{obs} and agonist concentration (see Figure 3A and B) and k_{off} estimates obtained from agonist washout experiments. (B) Correlation between k_{on} estimates obtained from the slope of linear fits to the relation between k_{obs} and agonist concentration and k_{off} estimates obtained from agonist washout experiments. Symbols and error bars represent means ± SEM. Note logarithmic scale on y-axis.

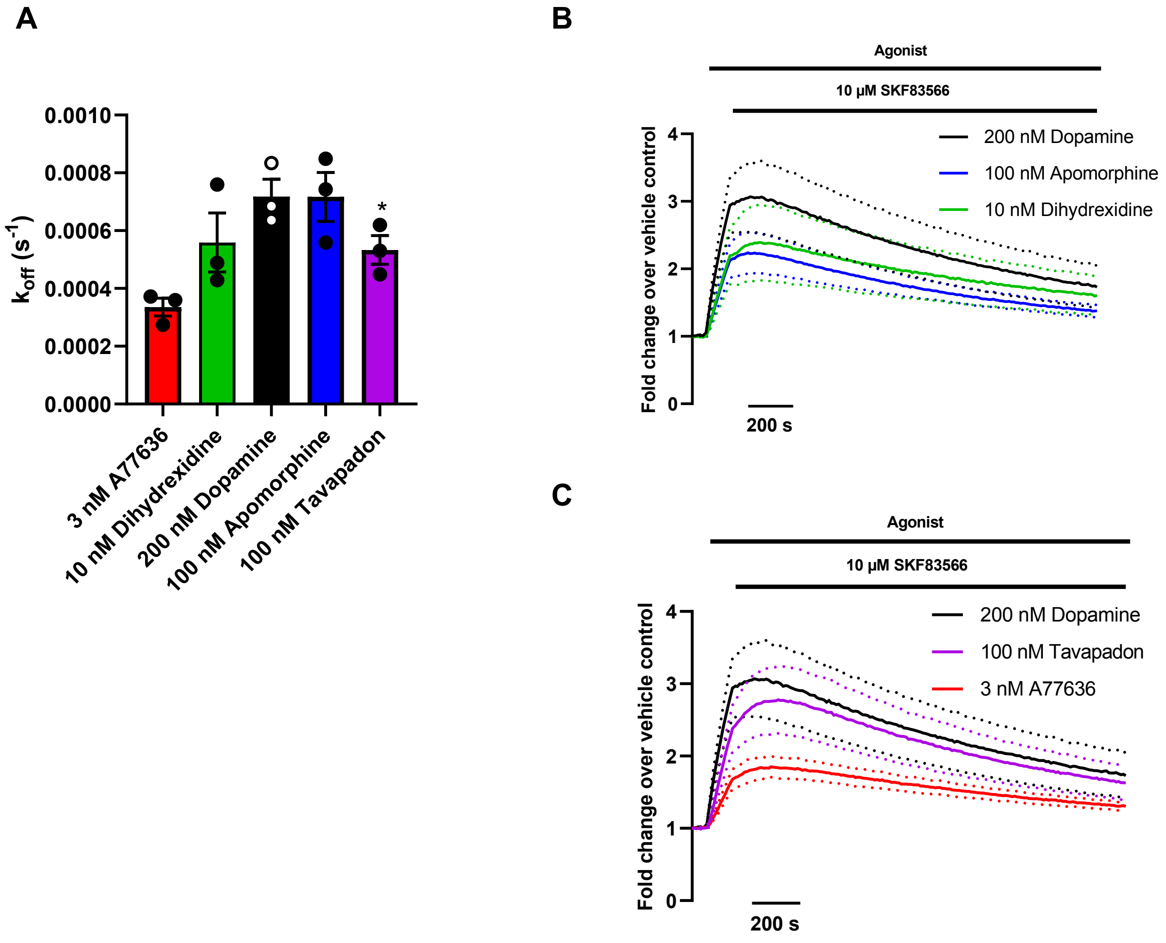

Supplementary Figure S3. Decay time courses of the response to D_1R agonists upon application of the D_1R antagonist SKF83566 to HEK293T cells expressing D_1R-NP in combination with LgBiT-miniG_s. A) k_{off} values derived from the fitted τs for response decay rate following SKF83566 application. Bars represent means ± SEM from three separate experiments performed in octuplicate wells and superimposed dots represent means from individual experiments. Asterisk indicates statistically significant difference from dopamine; *, $p < 0.05$, one-way repeated measures ANOVA with Dunnett's multiple comparisons test. B, C) Kinetic reads showing the luminescence increase following agonist application followed by luminescence decrease after addition of the D_1R antagonist SKF83566. For clarity, apomorphine and dihydrexidine (panel B) are shown separately from A77636 and tavapadon (panel C). Kinetic reads for dopamine are included in both panels for reference. Data points represent means (solid lines) ± SEM (dotted lines) from three to four separate experiments performed in octuplicate.
